# Barriers and facilitators to the introduction of import duties designed to prevent noncommunicable disease in Tonga: a case study

**DOI:** 10.1186/s12992-021-00788-z

**Published:** 2021-11-27

**Authors:** Colin Bell, Catherine Latu, Elisiva Na’ati, Wendy Snowdon, Marj Moodie, Gade Waqa

**Affiliations:** 1 Global Obesity Centre, Institute for Health Transformation, Deakin University, Geelong, Australia; 2 Pacific Research Centre for the Prevention of Obesity and Non-Communicable Diseases, WHO Collaborating Centre for Obesity Prevention and Management, Fiji Institute of Pacific Health Research (FIPHR), Suva, Fiji; 3 Public Health Division, Secretariat of the Pacific Community, Noumea, New Caledonia; 4 Deakin Health Economics, Institute for Health Transformation, Deakin University, Geelong, Australia

**Keywords:** Food policy, Nutrition, Tonga, Policy development, Import duty, non-communicable disease

## Abstract

**Background:**

In Tonga, import duties were lowered on tinned fish and seafood in 2013 and raised on soft drinks, dripping and other animal fats. Additional import duties were applied to soft drinks and dripping and other fats in 2016 and duties were also applied to high fat meats, mutton flaps and turkey tails. The objective of this study was to describe barriers to and facilitators of these import duties from a policy-maker perspective.

**Methods:**

A case study was conducted to analyse implementation of policies originally modelled by the Pacific Obesity Prevention in Communities project to reduce mortality in the Kingdom of Tonga. Policymakers (*n* = 15) from the Ministries of Revenue, Health, Finance and Labour and Commerce involved in the development and implementation of Tonga’s food-related policies participated in key-informant interviews.

**Results:**

The main facilitator of import duties were strong leadership and management, cross-sector collaboration, awareness raising and advocacy, nature of the policy, and the effective use of data to model policy impacts and inform the general public. The absence of clear lines of responsibility and a decline in collaboration over time were identified as barriers to implementation of the import duties.

**Conclusion:**

In a small Island state implementing import duties to prevent non-communicable disease can be straight forward providing policymakers and the community have a shared understanding of the health and economic costs of NCDs.

## Introduction

The Pacific Region is known for high prevalence rates of obesity and non-communicable diseases (NCDs) [1]. Rates are not only high, but are increasing faster in the Pacific than in the rest of the world [2]. Policies to improve food environments and prevent NCDs have strong evidence behind them [3], they are stated priorities across regional policy forums [4], and they align with World Health Organization recommendations [5]. However, fluctuations in political attention and inconsistences and tensions in sectoral responses frustrate comprehensive and sustained action [4]. A review of health services in the Asia-Pacific region indicates that the health systems response to NCDs, as well as the evidence base for program and policy development, remain insufficient [6].

The Kingdom of Tonga, one of 22 countries or territories in the Pacific Region, comprises 170 islands and a population of approximately 105,000. A study drawing on population surveys conducted in Tonga over the period 1973–2012, found that obesity prevalence increased from 56 to 70% and that type 2 diabetes mellitus increased from 5 to 19% [7]. In the early 2000s, adult mortality due to NCDs reduced life expectancy at birth in Tonga from 66 years for men and 69 years in women to between 60 and 64 years in men and between 65 and 69 years in women [8, 9]. This NCD burden has been attributed to a transition to processed foods in Tonga and other Pacific nations [10], with imported foods replacing demand for locally produced foods [11].

Tonga was the first country in the Pacific Region to establish a health promotion foundation (TongaHealth) for implementing NCD prevention interventions such as salt reduction [12]. It is also one of the first countries in the Pacific to embrace policy for improving diets to prevent NCDs [13]. Data modelling was undertaken to calculate the impact of 30 different food policy options, considered to be feasible and cost-effective, on mortality [14], and Tonga’s NCD strategy recommended that these options be adopted. The ramifications of the NCD burden for Tonga and the modelling data were brought to the political agenda through a meeting arranged between the Head of the Public Health Department and the Prime Minister in 2012 following a regional NCD forum. In response, a directive was issued to the Ministry of Revenue to form a cabinet approved taskforce committee to specifically focus on developing and implementing food-related fiscal policies. In August 2013, five fiscal policies increasing taxes on unhealthy food and decreasing taxes on healthy food were passed and became effective. In June 2016, further food taxes were implemented. Once again, a range of food products were taxed, and other ‘healthier’ products had taxes removed. This was endorsed by Cabinet with implementation on the 1st of July 2016. Tonga also included NCD targets and outcomes in their national Millennium Development Goals [15].

In line with the goal of the Australian National Health and Medical Research Council (NHMRC) Centre for Research on Obesity and Food Systems to support policy makers and public health advocates to create sustained policy change by evaluating potential policy options and their impacts on environments and systems [16], and with global efforts to understand how health policies emerge and are implemented in low and middle income countries [17], this paper explores the development and implementation of food related policy in Tonga. We describe national experience of health policy change and political and economic influences on policy with a view to informing future policy implementation and evaluation in Tonga and the Pacific [18]. A similar analysis has been completed for Fiji [19].

Research on health policy implementation typically presents general accounts of implementation experience or considers the views and experiences of implementing actors [20]. The aim of this paper is to present the views and experiences of policymakers in Tonga on barriers and facilitators to food policy implementation over the period 2013 to 2016.

## Methods

We adopted a case-study methodology. This allowed us to conduct an in-depth analysis using various sources of data (key informant interviews, government and other documents, academic literature), triangulated to tell a story [21, 22].

### Policy

The policies of interest were those originally modelled by Pacific Obesity Prevention in Communities project to reduce mortality in Tonga [14], and in particular, the fiscal policies subsequently adopted and implemented in 2013 and 2016. Data were collected in Tonga in 2016 and 2017.

### Participants and key informant interviews

Purposive sampling was used to identify policymakers involved in the development and implementation of Tonga’s food related policies. After obtaining endorsement from relevant permanent secretaries, government ministers from the Ministries of Revenue, Health, Finance and Labour and Commerce were invited to participate in key-informant interviews (KII). A snow-balling technique was used to recruit other policymakers until there was no new information or themes coming from the interviews. Informed consent was acquired before the interviews, which were conducted by EN and mostly in English. Interviews were audio-recorded and transcribed. Where the Tongan language was used, the transcriptions were translated into English and the translations were verified by a second researcher (CL) prior to analysis. Interviews were guided by a schedule developed to ascertain barriers and facilitators in the policy development process and designed to take 30 to 45 min to complete. The schedule was pre-tested by researchers to ensure the guiding questions were clear and the sequencing was logical. Ethics approval was received from the Tonga National Ethics and Research Committee and Deakin University Human Research Ethics Committee, Australia (HEAG-H 169_2014). Informed written consent was obtained from all participants and organisations.

### Document review

Formal government documents such as gazettes, budget statements, organisational policy guidelines and policy documents, as well as media reports, were reviewed for the 2013–2016 period. The review was conducted to confirm the progress and status of food-related policies and to triangulate the information provided in the key informant interviews [23].

### Analysis

We conducted a manifest content analysis to explore barriers and facilitators that were common to the food policies. Transcripts from the KII were checked against the audio-recordings for accuracy. Transcripts were then categorized, coded and verified against reviewed documents. Conflicting or contradictory statements were resolved in follow-up communication (email or phone) with informants. We did not explore evidence of absence. Themes were identified and analysed using Walt and Gilson’s policy triangle framework [24, 25]. Similar to the Fijian study, the analysis was also informed by theories related to the policy process and agenda setting. We drew on Kingdon to help identify policy windows (a confluence of factors contributing to policy change) and Sabatier to help identify patterns of interactions between actors [26, 27]. We provide quotes from key informants in line with the themes.

## Results

### Policy context

In 2009, staff from the Pacific Obesity Prevention in Communities (OPIC) project worked with local stakeholders in the Kingdom to identify 40 policy options that were potentially feasible and evaluated them, based on local data and global and regional evidence, for effectiveness and cost-effectiveness if implemented in the country [14]. A summary of the recommendations is presented in Table 1 along with implementation status and tax rates as of 2016. Because many food products are not domestically produced in Tonga and they are reliant on imports, several changes to import duties were recommended to reduce sugar and fatty meat and spread intake, and increase fish and vegetable intake. Excise duties were recommended when products were both imported and domestically produced to reduce soft drink, confectionary and fatty meat intake. Finally, new food regulations and changes to price controls were recommended to support these intake changes. While most recommendations were not implemented, legislation was enacted to increase fish consumption and reduce consumption of soft drinks and animal fats. In most cases, the implemented duties went further than the recommendation (eg reduction in import duty on tinned fish) and, importantly the mechanism used was import duties (including volumetric, weight based taxes) rather than excise duties, new food regulations or adjustments to price controls.

**Table 1:** Food policy recommendations for Tonga and implementation status as of 2016

OPIC Policy Recommendation 2011	Rationale	Implementation status
**Import duties**	Levied on goods from foreign countries	
Increase import duty from 0 to 15% for sugar	Reduce sugar intake	
Increase import duty from 0 to 15% for butter	Replacement with healthier fats	
Reduce import duty on fresh & frozen vegetables to 0%	Increase vegetable intake	
Reduce import duty on fresh fruits to 0%	Increase fruit intake	
Reduce import duty on tinned fish and seafood to 10% (fresh/frozen only those not caught locally)	Increase fish intake	Reduced from 20 to 5% in 2013
Reduce import duty tinned fish and seafood to 0%	Increase fish intake	
Reduce import duty tinned fish to 0%	Increase fish intake	
Reintroduce 15% import duty for high-fat meat and poultry (mutton flaps and turkey tails)	Replacement of high-fat meats with leaner meats	
Reduce import duty from 15 to 0% for margarine	Replacement of less healthy spreads	
Increase import duty corned beef/mutton from 0 to 15%	Replacement high-fat meats with leaner meats	
**Excise duties**	Levied on domestic goods	
Introduce 15% excise duty for soft drinks (all sweetened drinks including milk)	Reduce intake of soft drinks	Increased to $0.50/L in 2013 and $1/L in 2016
Introduce 30% excise duty for confectionary	Reduce confectionary intake	
Introduce excise duty of 15% for dripping and other animal fats	Replacement with healthier fats	Increased from 15% to $1/kg in 2013 and $2/kg in 2016
Introduce 15% excise duty for high fat meat/poultry	Replacement high-fat meats with leaner meats	A 40c/kg duty was introduced in 2016
Introduce 15% excise duty for corned beef/mutton	Replacement high-fat meats with leaner meats	
Introduce 15% excise duty for mutton flaps	Replacement high-fat meats with leaner meats	A 15% import duty was introduced in 2016
Introduce 50% excise duty for turkey tails	Replacement high-fat meats with leaner meats	A $1.50/kg duty was introduced in 2016
**Food regulations**	Protect health and safety	
Mandatory government workplace food policy	Healthier diets amongst government employees (increase fruit and vegetables)	
Removing license requirement for roadside vendors selling local fresh produce (uncooked, unprocessed) (to increase number of vendors)	Increase fish, fruit and vegetable intake	
Sales ban on high-fat meats	Replacement with lower-fat meats	
Regulation that processed meats sold contain no more than 20% fat	Replacement with lower-fat meats	
**Price controls**	Used to manage affordability of goods	
Add imported seafood to price control list	Increase fish intake	
Introduction of price control for bottled water	Reduce intake of soft drinks	
Imported fruits to be added to price control system	Increase fruit intake	
Add tinned fish to price control list	Increase fish intake	
Imported vegetables to be added to price control system	Increase vegetable intake	
Remove dripping from price control list	Replacement with healthier fats	
Remove unhealthy oils from price control list	Replacement with healthier oils	
Price control healthier meats	Replacement of higher-fat meats with healthier ones	
Remove cheese from price control list	Decrease cheese intake, replacement with lean meat or fish	

Adapted from Snowdon et al. [14]

### Policymakers

Key informant interviews were conducted with 15 participants from the Ministries of Revenue, Health, Finance and Labour & Commerce. One key informant passed away. From these interviews, five themes were identified as barriers to and/or facilitators of food-related policy in Tonga: leadership and management; cross-sector collaboration; awareness and advocacy; the nature and intent of the policy; and the use of evidence. These are expanded on below and illustrated with quotes from policymakers.

### Leadership and management

Key informants were of the view that the absence of identified leaders for implementing the recommendations hindered progress. All the recommendations were advocated for and well received by the Ministry of Health as evidenced by the NCD strategy recommending government action. However, it was unclear who was responsible for implementation.

*“The policy proposal was actually advocated for by the Public Health [CMO PH] for a long time. Unfortunately for some reason, the enthusiasm by [CMO PH] was not followed through.”*

*“ … we did not also look at the body that will implement the policies and to make specific timelines and key responsibilities so they can tie down the key stakeholders to clearly state this is the deadline we’re working towards and do 1, 2, all the way through to 40 … ”*

*“It was important that we identified the needs but moving on to processing policies, we are not sure of who was supposed to do it, whether it was Health or us or any other...”*

One informant suggested that more of the recommendations may have been taken up if responsibility for their passage to and through parliament was assigned to particular institutions and individuals. Another informant implied that the lack of clear lines of responsibility contributed to a gap in the pathway from translating evidence into policy action.

*“ … even when it’s already published [OPIC recommendations]. It should be included in our routine advocacy materials … there is a big gap from evidence and research to policy intervention … ”*

Secondly, informants noted a ‘lack of enthusiasm by senior health officials’ as another leadership-themed barrier to implementation. This was attributed to the novelty of food-related fiscal policies in Tonga and an associated fear of taking action. Also, to a lack of knowledge about trade in the health ministry and memories of an unsuccessful attempt to place an import quota on mutton flaps that compromised World Trade Organisation accession plans [28].

*“ … I think people don’t have the courage to move outside of their comfort zone. Most of us like to be followers, we wait for others to do it first … ”*

“ … *there was not enough trade expertise within health and there was not much discussion about trade. But I think there was a perception that it will be very difficult. So, no one really ventured into these kind of things … ”*

On the flip side, incisive individual leadership was identified as the reason for the 2013 fiscal policy changes being implemented so quickly. The determination of the then Chief Medical Officer, Public Health (CMO PH) resulted, not only in a face-to-face meeting with the Prime Minister of Tonga, but a directive to the Minister of Revenue to fast track the policy development process.

*“ … it didn’t go through the [normal] process. He used his own line of communication to achieve political intervention and we see how strong that political intervention [was] … ”*

Good organisation leadership from the Minister of Revenue, was given as the explanation for the smooth and fast-tracked implementation of the 2016 food taxes. The Minister instructed staff in the Revenue and Customs Ministry to analyse import revenue data, model the proposed food- related taxes scenarios, discuss them with senior staff and adjust taxes and/or select other food products based on the scenarios. The legal section within the same Ministry then drafted the amendment for submission to Cabinet. In Tonga, once a tax policy is endorsed, it becomes effective immediately. There was some push-back from business, particularly to the sugar-sweetened beverage excise duties, that led to the Ministry of Revenue demonstrating leadership and enforcing the law.

*“This is a government decision, there are times when we need to consult with [businesses] but there are times when we [government] need to make our own decision”.*

*“In relation to the SSB tax, the industry said ‘we have already put our orders through’ but the response from [government] was ‘that is actually not our responsibility, you still have to pay what the rate is now’”*

### Cross-sector collaboration

The Prime Minister’s directive to form a task force committee in 2013, led by the Ministry of Revenue and attended by high level representatives from Commerce, Health, Revenue and Finance, expedited endorsement of the 2013 NCD-related taxes. According to the informants, this cross-sector collaboration ensured relevant Ministries had input on the policies, that decisions were debated and made, and that feedback was provided to Ministers.

*“It has been teamwork, a coordinated effort between the different ministries – health, finance, revenue and customs, and labour. No one can do it without the support of the other. We need to remember the partnership, the people and the politicians, to work together, and the champions who willingly did it without recognition or need for acknowledgement. We know it is for the best of the country.”*

Decisions on which of the proposed tariff rates to change made at these meetings were informed by research provided by the World Health Organization and data provided by the Ministries of Revenue and Commerce. To minimise potential opposition to the proposed tariffs, the task force also made a strategic decision to “start small” with a plan to expand the range of food products that incurred excise taxes and progressively increase taxes on certain products. This was the reason behind the two-tiered introduction of taxes in 2013 and 2016.

*“We decided that, in order to be successful with this policy, we had to start small, and that is why only 3 items were initially selected.”*

While the need for collaboration was diminished in 2016 as higher taxes had already been flagged in the progression plan, participants noticed a lack of cross-sectoral collaboration the second time around. In particular, there was a pause in collaboration between the Ministries of Revenue and Health.

*“We hoped to be working together with Health – I approached them [Health] but we got sick of waiting... But now it’s good … It’s an ongoing process.”*

*“We still need the support of the Ministry of Health and their attendance at the committee.”*

The Ministry of Health were of the view that the Ministry of Revenue should initiate action because tax was the focus of the policies being implemented. It saw the Ministry of Revenue’s role in the policy implementation process as supporting the health argument underpinning the policy.

*“We have raised that concept of health in all policies. For us, we are quite aware 70% of determinants of health happens outside of health … for food we put it out to Ministry of Agriculture. When it comes to taxation, we send it to Revenue to be involved in the development of policies. Most of the excise tax initiative was by Customs and Revenue. When they go public, we provide the support to make sure it’s not a money-making thing but a health gain process.”*

### Awareness and advocacy

Growing global and national awareness of the health and economic burden of NCDs in the leadup to the introduction of the 2013 food-related taxes was identified by participants as important to them being passed. In 2011, the High Level Meeting in New York on NCDs set a platform for NCD work to move forward. Soon after, Tonga conducted its second NCD Risk Factor STEP Survey in 2011/2012 with NCD screening being conducted with a representative sample of Tongans across the nation. Both activities raised awareness of Tonga’s NCD burden among civil society and policymakers and created a favourable environment for endorsement of the proposed taxes. Furthermore, national documents articulated the need for NCD action. The Tonga National Strategic Framework 2011–2014 included a specific objective to address the rise in NCD burden within the country. This was further supported by a National NCD Strategic Plan that looked at strategies to overcome some of Tonga’s worst NCD indicators. Collectively raised awareness and clear strategic directions ensured the proposal to tax unhealthy foods and make heathy food affordable was seen as a “win-win”, good for health and able to generate revenue.

*“There was great support for this to be implemented. But I must say, there is still a lot of work to be done in terms of continuing this fight against NCD.”*

The CMO PH was recognised by informants as the advocate for the ‘fight against NCDs’ and the taxes and one informant noted that this continued following the introduction of the 2013 laws. The Chief Medical Officer went on national television to encourage compliance with the laws and to publicly acknowledge the contribution of policymakers.

*“That was good that he did that because we would not have gone public ourselves.”*

Informants indicated that there was much less awareness raising or advocacy in the lead up to the introduction of the 2016 taxes. Also, in keeping with the lack of consultation mentioned previously, there was no implementation task force. The taskforce committee was bypassed, and the taxes were simply implemented by the Ministry of Revenue. While this didn’t hinder implementation, informants noted that there were a number of complaints about the 2016 taxes and this ‘push-back’ appeared in the media.

### Nature and intent of the policy

The 2013 and 2016 policy changes were in keeping with Tonga’s existing tax schedule and tariff bands. Informants noted that this made policy endorsement easier. Brand new policy would have required extensive consultation, more drafting responsibilities and greater buy-in from policymakers. Informants also noted that the policies targeted food products (like lard or dripping) that were unlikely to invoke strong public opposition. If the tax had been applied to chicken, they did not think the policy would have been endorsed.

*“Meat is more expensive in Tonga than Fiji (e.g. chicken is $10 per kilo but in Fiji it is $4 per kilo), but fruit and vegetables are cheaper. The committee was told not to touch chicken [change the excise duty on it] because people will not be happy.”*

Later in the policy process a decision was made to tax chicken quarters but, in response to public pushback and evidence that it would have a regressive impact on low-income households, it was removed [29]. There was also some concern that the sugar-sweetened beverage tax may not have been endorsed due to public and industry opposition.

*“[Sugar-sweetened beverages] could have been a huge issue, but at the end of the day it was approved.”*

*“At least we succeeded with the sugar sweetened drinks because, as you know, in our culture we use a lot of them eh. We not only love it, but we use the drinks at funerals”*

The fact that they were fiscal policies with the capacity to generate revenue also made their endorsement easier. Policymakers noted that in the context of slow economic growth for Tonga, plans to host the South Pacific Games (which were subsequently cancelled), and decreased foreign allocation from developing partners, it was the revenue generating nature of the policy that most likely clinched the endorsement of cabinet members and the Prime Minister.

Finally, timing in the political cycle and the number of policies under consideration at any one time were also noted as factors influencing policy endorsement.

*“You wouldn’t want to do it [introduce new taxes] 1 year before the election. Most of the people won’t support it because they don’t want to pay more tax.”*

*“You have to be sensitive to the political climate and know when to implement the change.”*

*“He was scared that they were all there.” [that there were too many policy recommendations for cabinet to consider all at once]*

It was noted that a champion of the policies became sick at a critical time in the policy endorsement process and that other policy recommendations may have been endorsed had that not happened.

### Use of evidence

Information gathering for 2013 policies was performed by a research team within the Ministry of Revenue and Customs. They sourced health data from WHO, employment and economic data from the Ministry of Labour and Commerce and import data from the Ministry of Revenue. Informants considered this data, and its subsequent collation and presentation to the committee, as critical for determining what products were to be taxed and by how much.

*“He did a lot of work and sourced data from labour. The process was hard and took a long time. We needed the support of people writing the papers and getting the research done.”*

According to informants, the taxes were designed to be progressive with a ‘soft’ introduction and increases over time. It was noted that this was dependent on successive governments being in agreement.

*“We wanted the tax to be progressive but, in the end, we left any subsequent increases to other governments.”*

Illustrating the importance of government conducting their own research, one policymaker made the following observation about the proposed tobacco tax, which was being considered at the same time as the food taxes.

*“They [the tobacco industry] were proactive about the tobacco tax and even asked for an increase of 5%. We said ‘no, no, no we can’t do what you say, we have to do our own analysis’. The tobacco industry has a contact in Tonga who is always ringing me to have a meeting, it is always like that. At the end of it, we have to do our own work to ensure that we actually don’t do what they want.”*

Informants did not identify evidence-use barriers to implementation.

## Discussion

Tonga experienced swift and seamless endorsement of import duties on soft drinks and fatty meats to prevent non-communicable diseases in 2013 and 2016. In 2013, proposals went through comprehensive discussions and consultations with relevant stakeholders and a taskforce committee was appointed to deliberate on decisions regarding which products to tax. In 2016, benefiting from the groundwork in 2013, one Ministry was in charge of policy formulation and development and successfully carried several new import duties through to endorsement. Through both processes, leadership and management, cross-sector collaboration, awareness raising and advocacy, the nature of the policy and use of evidence facilitated the implementation of these duties.

Similar facilitators were found for the policy development process in Fiji [19]. Fiji considered a number of policy recommendations designed to reduce the population’s exposure to unhealthy foods and increase the availability of healthy foods. Import duties were reduced on imported vegetables and fruits [30], increased on palm oil [31], and education policy on food sold in school canteens was strengthened. Policymakers in Fiji identified leadership and specific leaders as critical for getting policy proposals on the government’s agenda and noted, as Tongan policymakers did, that where this was absent, proposals (such as the one to reduce unhealthy marketing to children) did not progress. They also reported that collaboration between sectors, the nature and content of the policy, and the timing of the passage of proposals through parliament influenced the likelihood of endorsement. Drawing on findings from Fiji and Tonga, our observation is that the greater the financial impact a duty is likely to have and the higher the status of the food being considered for taxation, the stronger the argument needs to be, the more collaboration and leadership required and the more critical the timing. What the findings from Tonga add to this observation is the importance of awareness and advocacy. In Tonga, raising awareness of the burden of NCDs and linking this directly to the tax increases meant everyone understood why they were being implemented. The results from Tonga also highlight the importance of robust and independent research for tax proposals [31].

A systematic review and meta-synthesis of international studies of obesity prevention policy underpinned by political science theories describes similar influences on policy decision-making [32]. One influence identified in the review was personal values and beliefs with the authors noting that the motivations behind the actions of key individuals and leaders were linked to their experiences, values, beliefs and political ideologies. While not an obvious theme in our study, it was clear that personal values came into play through the chief medical officer and through the shared understanding of policymakers that the taxes would benefit health. It should be noted that the studies included in the review were all from high income countries, namely the UK, Canada and predominately the USA. The difference between these countries and developing countries such as Tonga has been described by Peters as “the degree of difficulty governments encountered in the policymaking process” with Peters going on to state that “less developed countries may actually enjoy some real advantage” [33]. He attributes this potential advantage, in part, to less resistance to change and innovation given less “solidified” policy making systems and the belief by developing countries that they have much to learn from developed countries. We concur that Tonga has several policymaking advantages over a country like the USA and attribute this to smaller government and fewer competing priorities. Also, rather than learning from developed countries, our view is that Tonga and other developing countries see the opportunity to lead the world in regulatory approaches to NCD prevention. One challenge that will continue to be faced by Tonga and similar small island states however is the power of large countries and organisations to dictate terms of trade. As the mutton flap quota mentioned earlier illustrates, a national policy is redundant if it can’t be implemented.

Have the import duties worked? Encouragingly, since endorsement of the soft drink import duties, there is evidence they have had the intended impact on price and imports. Sourcing import and revenue data from Tonga customs and conducting a time series analysis, Teng et al., report that the prices of indicator soft drinks have increased and that import volumes decreased in line with tax increases in 2013, 2016 and 2017 [34]. Also, there is some evidence that low-income households experienced greater declines in soft drink expenditure than high-income households [35]. However, sales of domestically produced soft drink increased demonstrating that import duties have translated to increased consumption of locally produced foods as envisaged by Snowdon et al. (2011) [14], but not towards healthier foods. A 2017 study commissioned by FAO used similar methods to this study to explore stakeholder perspectives on the effectiveness of fatty-meat taxes in Tonga [36]. Stakeholders, who included participants (*n* = 20) from government ministries and grocery stores as well as consumers (whose views were recorded in focus groups), reported increased prices and decreased purchases of mutton flaps and turkey tails but no change in purchasing of imported fatty, chicken quarter legs. Fish was recognised as a healthier alternative to chicken legs (and duties were reduced in 2013), but consumers reported that tinned fish was still more expensive than chicken. Further research is needed to explore the health impact of food-related policy in Tonga. The government of Tonga continues to progress taxation alongside other measures to prevent NCDs. In collaboration with the World Bank, they have developed and endorsed an evidence-based, non-discriminatory nutrient profile model to inform and scale up taxation.

### Strengths and limitations

A strength of this study is the depth of insight provided by policymakers involved in the policy endorsement process. Also, we were able to triangulate their insights with government documents and academic literature. The theoretical frameworks were helpful for ensuring we captured critical elements of the policy making process although, because they were developed in European and North American contexts, they didn’t always align with the Tongan context. Limitations include our inability to interview all policymakers who contributed to the process because of refusal or unavailability and, the possibility that those who were interviewed did not feel free to speak their minds. Participants remained anonymous but they may still have been concerned given the small number of people involved. Finally, the manifest content analysis allowed us to examine exactly what was said in the interviews but, given the prominence of oral traditions in Tonga and even with Tongan interviewers, we may have missed intended meanings.

## Conclusion

This case study captured the views and experiences of policymakers in Tonga on barriers and facilitators to reducing import duties on fish and increasing duties on soft drinks and fatty meats in Tonga over the period 2013 to 2016. Policymakers described a mix of leadership and management, cross-sector collaboration, awareness raising and advocacy and use of evidence that helped them avoid barriers and culminated in the endorsement and implementation of the duties. The findings are relevant for other countries, particularly small island developing states with similarly high dependence on imported foods and high non-communicable disease prevalence.

## Data Availability

Transcripts are available for authors on request.
